# Safety and Pharmacokinetics of Long‐Acting Monoclonal Antibodies Tixagevimab and Cilgavimab (AZD7442) in a China Phase 2 Study and Evaluation of Asian Race Effect

**DOI:** 10.1002/cpdd.1586

**Published:** 2025-09-05

**Authors:** Jing Zhang, Huixia Zhang, Yajuan Zhang, Shuyuan Liu, Xiaoyun Ge, Haiyue Zhang, Yunfei Li, Cecil Chi‐Keung Chen, Oleg Stepanov, Weifeng Tang, Wenhong Zhang

**Affiliations:** ^1^ Clinical Trial Institution Clinical Pharmacology Research Center Huashan Hospital Fudan University Shanghai China; ^2^ Clinical Pharmacology and Quantitative Pharmacology Clinical Pharmacology & Safety Sciences BioPharmaceuticals R&D AstraZeneca Gaithersburg MD USA; ^3^ Respiratory & Immunology R&D China AstraZeneca Shanghai China; ^4^ Clinical Safety R&D China AstraZeneca Shanghai China; ^5^ Biometrics R&D China AstraZeneca Shanghai China; ^6^ R&D China AstraZeneca Shanghai China; ^7^ Clinical Pharmacology and Safety Sciences BioPharmaceuticals R&D AstraZeneca South San Francisco CA USA; ^8^ Clinical Pharmacology and Safety Sciences BioPharmaceuticals R&D AstraZeneca Cambridge UK; ^9^ Department of Infectious Diseases Huashan Hospital Fudan University Shanghai China; ^10^ Shanghai Sci‐Tech Inno Center for Infection & Immunity Shanghai China

**Keywords:** clinical trial, COVID‐19, monoclonal antibody, pharmacokinetics, race effect

## Abstract

Safety, pharmacokinetics, and impact of race of pharmacokinetics on monoclonal antibodies tixagevimab and cilgavimab (AZD7442) were assessed in Chinese adult participants in a Phase 2, randomized, double‐blind, placebo‐controlled trial. In total, 272 participants were randomized 3:1 to a single intravenous dose of 600 mg AZD7442 or placebo and followed for 451 days. Mean participant age was 34.2 years, 5.9% were aged greater than 60 years, and 69.1% were male. Adverse events (AEs) occurred in 72.8% and 80.0% of participants with AZD7442 and placebo, respectively; most were mild or moderate in severity. Serious AEs were reported in 3.0% and 4.3% of participants with AZD7442 and placebo, respectively. No AEs of special interest, infusion‐related reactions, or deaths occurred. Maximum serum concentrations of tixagevimab and cilgavimab were rapidly achieved following infusion, then declined through Day 361. Mean half‐lives were 85 days for tixagevimab and 80 days for cilgavimab. AZD7442 recipients exhibited greater than 4‐fold neutralizing antibody titer increases versus baseline at Day 8, which then declined through Day 361. Among AZD7442 recipients, 20.8% were treatment‐emergent antidrug antibody positive. Asian race had no clinically significant impact on AZD7442 pharmacokinetics. Overall, intravenous 600 mg AZD7442 was well tolerated in Chinese adult participants. AZD7442 pharmacokinetics were similar in Asian and non‐Asian participants.

**ClinicalTrials.gov identifier**: NCT05184062

Although no longer considered a global health concern, COVID‐19 continues to be a threat, particularly for immunocompromised individuals who have suboptimal responses to vaccines and continue to experience severe disease, hospitalization, and death due to COVID‐19.^1^, ^2^, ^3^

AZD7442 consists of 2 monoclonal antibodies (mAbs), tixagevimab and cilgavimab, that bind to distinct sites on the severe acute respiratory syndrome coronavirus 2 (SARS‐CoV‐2) spike protein receptor‐binding domain.^4^ The mAb components were modified with YTE and TM amino acid substitutions to extend half‐life and thus duration of protection and reduce the potential risk of antibody‐mediated enhancement of disease, respectively.^4^ A Phase 1, first‐in‐human study investigating AZD7442 in healthy adult participants showed that AZD7442 was well tolerated, with a favorable safety profile across all intramuscular (300 mg) and intravenous (IV) (300‐3000 mg) doses.^5^ Furthermore, tixagevimab and cilgavimab had similar pharmacokinetic (PK) profiles, and SARS‐CoV‐2‐specific neutralizing antibody titers increased in a dose‐dependent manner in parallel with AZD7442 concentration and were maintained above the level of convalescent plasma throughout the 361‐day study period.^5^ In Phase 3 studies, AZD7442 demonstrated efficacy for the prevention and treatment of COVID‐19, with favorable safety outcomes.^6^, ^7^ However, AZD7442 showed loss of neutralization activity against Omicron variants including BQ1.1 and XBB.^8^, ^9^ However, as AZD7442 represented the first long‐acting mAbs to be authorized for preexposure prophylaxis of COVID‐19, new data from clinical trials are still important to inform development of future long‐acting mAbs using the same YTE/TM modifications as AZD7442, for prevention of COVID‐19 and other infectious diseases.

It is important to assess the safety and pharmacology of new therapeutics in a diverse population to ensure representativeness of the results. However, the global Phase 3 studies of AZD7442 recruited few participants of Asian race,^6^, ^7^ although a subsequent Phase 1 study assessed AZD7442 safety in PK in Japanese participants.^10^ Although no longer available for clinical use, assessment of the safety and PK of AZD7442 in populations underrepresented in global clinical trials may help inform development of future mAbs containing the same YTE/TM modifications as AZD7442. Here, we report results from a Phase 2 randomized, placebo‐controlled study that evaluated the safety, tolerability, and PK of IV 600 mg AZD7442 administered to Chinese adult participants, including those with stable medical conditions. In addition, the potential impact of race on AZD7442 PK was evaluated using a population PK approach.^11^

## Methods

### Study Design

This Phase 2, randomized, double‐blind, placebo‐controlled study (ClinicalTrials.gov: NCT05184062) evaluated the safety, tolerability, PK, and pharmacodynamics (PD) of AZD7442 in Chinese adult participants aged 18 years or older, including healthy participants and those with stable medical conditions. This study was conducted in 14 clinical study centers in mainland China (Table S1).

This study was performed in accordance with the ethical principles that have their origin in the Declaration of Helsinki and that are consistent with International Council for Harmonisation guidance, Good Clinical Practice, applicable regulatory requirements, and AstraZeneca policies on bioethics and human biological samples. The protocol was reviewed by the relevant institutional review board/ethics committee at the respective study sites (Table S1). All participants provided informed written consent.

The study had 2 planned analyses. The primary analysis was conducted after all treated participants completed (or withdrew from) follow‐up through Day 181. The final analysis was conducted after all treated participants completed the study (ie, completed follow‐up through Day 451) or withdrew from the study. The first participant was enrolled on December 3, 2021. The primary (6‐month) analysis data cutoff was on August 15, 2022. The last participant visit was on May 6, 2023. Results are reported from the final analysis (completed on July 14, 2023).

Participants were randomized 3:1 to receive a single dose of either IV 600 mg AZD7442 or placebo. Randomization was stratified by age (greater than 60 or 60 years or younger) and COVID‐19 vaccination status (vaccinated or unvaccinated) at screening. The study was expected to be approximately 479 days in duration for each participant, consisting of a screening period of up to 28 days (Days –28 through –1), an intervention period of 1 day (Day 1 [randomization and dosing]), and a safety follow‐up period of 450 days (Days 2 through 451).

### Study Treatments

Tixagevimab and cilgavimab were provided in separate vials as sterile, clear to opalescent, colorless to yellow solutions. The solutions contained 100 mg/mL of active ingredient (tixagevimab or cilgavimab) in 20 mM of L histidine/L histidine hydrochloride, 240 mM sucrose, and 0.04% (w/v) polysorbate 80, at pH 6.0. Placebo was provided as a sterile solution of 0.9% (w/v) sodium chloride.

For IV administration, tixagevimab and cilgavimab were added to a single IV bag and gently mixed before infusion. The 600‐mg IV infusion comprised 3 mL of tixagevimab plus 3 mL of cilgavimab and the target infusion time was 30 minutes (20 mg/min). There was no specific guidance on the administration site for IV infusions, and this was not recorded, although it is typically the left or right hand in clinical practice.

### Participants

For inclusion in the study, participants had to be aged 18 years or older at the time of signing the informed consent; healthy by medical history, physical examination, and baseline safety laboratory tests, or had stable medical conditions that could benefit from passive immunization with antibodies, with negative results of SARS‐CoV‐2 quantitative reverse transcription polymerase chain reaction (qRT‐PCR) test within 14 days before randomization; and able to complete the follow‐up period through Day 451.

Stable medical conditions were defined as no hospitalization or emergency visit for worsening of disease/condition within the 12 months before enrollment, no acute change in the participant's condition at the time of study enrollment, and no exacerbation in disease/condition and no significant change in therapy expected during at least the first 6 months of the study. Medical conditions included risk factors for COVID‐19 or risk factors for suboptimal responses to vaccines, including age 60 years or older, body mass index 30 kg/m^2^ or greater, chronic obstructive pulmonary disease, congestive heart failure (New York Heart Association classification class II or lower), chronic kidney disease, chronic liver disease, immunocompromised state requiring maintenance use of corticosteroids and/or other immunosuppressive medicines, vaccine intolerance, or any other disease. Participation in the study was not expected to pose a significant risk to the participant. Full eligibility criteria are listed in the Supplemental Information.

### Randomization and Blinding

All participants who fulfilled the eligibility criteria were centrally assigned to the randomized study intervention using interactive response technology/randomization and trial supply management. Investigators and participants remained blinded to the assigned study intervention until the end of the study. Selected personnel from the sponsor were unblinded after the primary clinical data lock to perform the analysis for the report of the primary analysis.

### End Points

Safety assessments included adverse events (AEs), serious AEs, and AEs of special interest (AESIs). The prespecified AESIs in this study were anaphylaxis and other serious hypersensitivity reactions, including immune complex disease and infusion‐related reactions.

Also assessed were serum concentrations of tixagevimab and cilgavimab, antidrug antibodies (ADAs) against tixagevimab and cilgavimab, neutralizing responses against SARS‐CoV‐2 in serum, SARS‐CoV‐2 qRT‐PCR test for participants with symptomatic COVID‐19, and postbaseline SARS‐CoV‐2 serology tests. PK parameters were derived using noncompartmental analysis methods. Serum samples for neutralizing antibody titers against SARS‐CoV‐2 were collected on Study Days 1, 8, 31, 61, 91, 181, 271, and 361.

Treatment‐emergent ADAs (TE‐ADAs) were defined as those occurring in participants who were ADA negative at baseline but ADA positive after baseline, or with a 4‐fold or greater increase in ADA titer after baseline (see further details in Table S2 note).

A SARS‐CoV‐2 qRT‐PCR test was performed at the discretion of the investigator if the participant met 1 or more of the following qualifying symptoms: fever, shortness of breath, or difficulty breathing (all of no minimum duration) or chills, cough, fatigue, muscle aches, body aches, headache, new loss of taste, new loss of smell, sore throat, congestion, runny nose, nausea, vomiting, or diarrhea (all must be present for 2 days or more). qRT‐PCR tests had to be performed at the central laboratory to be included in the analysis.

The SARS‐CoV‐2 serology tests were performed on Days 1, 181, and 361. To be considered postbaseline positive in the end point analysis, the participant had to have a positive result from the validated assay performed at the central laboratory. Where data were available, the proportion of participants with a posttreatment response (negative at baseline to positive following treatment with study intervention, excluding data after on‐study vaccination, if any) for SARS‐CoV‐2 antibodies was summarized by intervention group and study total.

#### Assays

Details of methods for measuring serum tixagevimab and cilgavimab concentrations, SARS‐CoV‐2 neutralizing antibodies, and ADAs are reported separately.^12^

### Statistical Analysis

The planned sample size was 272 participants. If the true AE rate was 1% at the primary time point (ie, Month 6), 162 participants in the AZD7442 group would provide a probability of 80% or greater to observe 1 or more AE cases.

Baseline and safety data were analyzed in the safety analysis set, which included all participants who received AZD7442 or placebo. Erroneously treated participants were analyzed according to the intervention they received. PK analyses were conducted in the PK analysis set, which included all participants in the safety analysis set who received AZD7442 and had 1 or more quantifiable serum PK observations after dosing, with no important protocol deviations thought to impact the analysis of the PK data. Similarly, ADA analyses were conducted in the ADA analysis set (all participants in the safety analysis set with a nonmissing baseline ADA result and 1 or more nonmissing postbaseline ADA results), and PD analyses were conducted in the PD analysis set (all participants in the safety analysis set with 1 or more quantifiable titer observations after dosing, with no important protocol deviations thought to impact the analysis).

Baseline and safety data were presented using descriptive statistics. All statistical computations were performed using SAS Version 9.4 (SAS Institute Inc.), or higher. PK parameters were derived using noncompartmental analysis methods with Phoenix WinNonlin Version 8.1 (Certara Inc.) or higher. AZD7442 serum concentrations represent the arithmetic sum of tixagevimab and cilgavimab concentrations. Serum concentration values below the lower limit of quantification were set to 0 for inclusion in the calculation of means.

### Effect of Race on PK

To assess whether there are any differences in AZD7442 PK in different race groups, the serum PK parameters of tixagevimab and cilgavimab after IV infusion of AZD7442 were summarized from 3 clinical trials in Asian populations (Phase 1 and 2 trials including participants from China [NCT05437289; NCT05184062] and a Phase 1 trial including participants from Japan^10^) and a Phase 1 trial in a global population^5^ (including 68% White, 14% Asian, and 8% Black participants, and 10% of other race groups).

The effect of Asian race on AZD7442 PK was also explored in a population PK analysis, whereby participants in the Japan Phase 1 and China Phase 1 and 2 trials were pooled with Asian participants from 5 other global studies as 1 covariate (Asian race) in an AZD7442 population PK model.^11^ Further details regarding the population PK analysis methods, including model building and evaluation, have been reported previously.^13^ To assess the independent impact of Asian race, covariate searches were conducted in a model that included Asian race as a covariate and accounted for body weight. Body weight was included a covariate for clearance (CL) and volume of distribution in the model. The typical CL value was based on a typical participant, defined by a demographic summary (ie, median for continuous covariates or most prevalent category for categorical covariates): baseline weight, 70 kg; female sex; age, 65 years or younger; body mass index, <30 kg/m^2^; no diabetes; and non‐Asian race. Simulations were performed 10,000 times for the typical participant and participants with body weight 55.4 kg, body weight 122 kg, and Asian race, to account for standard error in parameter estimation for CL and covariate effects. A covariate effect was considered not clinically relevant if the absolute relative change in the parameter, induced by the covariate at either the 5th or 95th percentile of a continuous covariate (or at specific levels for a categorical covariate), was less than 20% of the typical value.

## Results

All results are presented from the final analysis.

### Participant Disposition

In total, 726 participants were screened, of whom 272 participants were randomized 3:1 to IV 600 mg AZD7442 (N = 202) or placebo (N = 70) (Figure S1). All 272 randomized participants completed the assigned study treatment. The most common reason for failing the screening period was not meeting the entry criteria (N = 390). Overall, 265 (97.4%) randomized participants completed the study through to Day 451 follow‐up. Only 7 (2.6%) randomized participants withdrew from the study. Five participants in the AZD7442 group and 1 participant in the placebo group were lost to follow‐up; 1 participant in the AZD7442 group withdrew consent. The median duration of follow‐up was 447 (range, 149‐461) days in the AZD7442 group and 448 (range, 336‐456) days in the placebo group.

### Baseline and Demographic Characteristics

Baseline and demographic characteristics were well balanced between treatment groups (Table S3). The mean age of participants was 34.2 years. All participants were Asian, 5.9% were aged greater than 60 years, and 69.1% were male. In total, 51.5% were COVID‐19 vaccinated (ie, 6 months or more before randomization). The study participants were representative of the target population for this study: healthy adults or those with stable medical conditions in China.

### Safety

AEs were reported in 147 (72.8%) participants with AZD7442 and 56 (80.0%) participants with placebo (Table S4). Most AEs were mild or moderate in intensity. In the AZD7442 group, the most common AEs (more than 5%) were COVID‐19 (28.7%), upper respiratory tract infection (14.4%), influenza‐like illness (9.9%), protein urine present (7.4%), and suspected COVID‐19 (5.9%). In the placebo group, the most common AEs were COVID‐19 (28.6%); suspected COVID‐19 (20.0%); upper respiratory tract infection (18.6%); protein urine present (8.6%); and influenza‐like illness, headache, and nasopharyngitis (5.7% each) (Table S4).

Overall, COVID‐19‐related AEs were reported in 71 (35.1%) participants in the AZD7442 group and in 34 (48.6%) participants in the placebo group. For the majority of participants with COVID‐19–related AEs (103 of 105 participants), the onset date of the first COVID‐19‐related AE was late in the study (with onset dates from Day 278 onward).

Serious AEs were reported in 3.0% and 4.3% of participants in the AZD7442 and placebo groups, respectively. Drug‐related AEs were reported in 9.4% and 10.0% of participants in the AZD7442 and placebo groups, respectively. No deaths occurred. There were no AESIs (ie, serious hypersensitivity or infusion‐related reactions) in the study. Two (1.0%) participants reported an AE (infusion‐site swelling and infusion‐site edema) leading to dose interruption in the AZD7442 group; both events were considered by the investigator as related to the study procedure and not to the study drug.

### Pharmacokinetics

**Figure 1:**
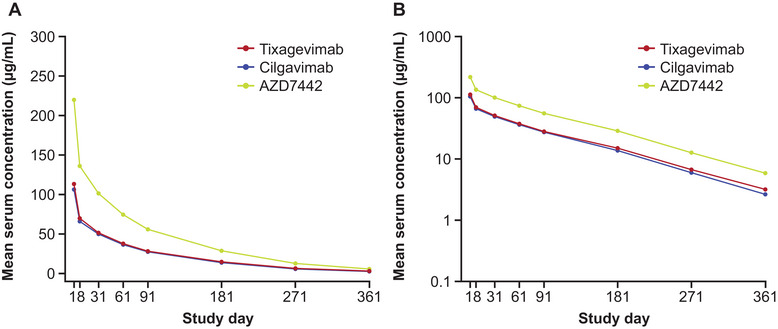
Serum concentration (µg/mL) of tixagevimab, cilgavimab, and AZD7442 (tixagevimab/cilgavimab) versus time plotted on linear (A) and semilogarithmic (B) scales (PK analysis set). Results represent arithmetic mean serum concentrations after administration of IV 600 mg AZD7442 (n = 202). Nominal sampling elapsed time (ie, time difference between nominal sampling time and dosing time) is used for mean summary plots. Predose time equals 0. The concentration value of AZD7442 is the arithmetic sum of tixagevimab and cilgavimab concentration values. There were no values below the lower limit of quantification (0.3 µg/mL). PK, pharmacokinetic.

Arithmetic mean serum concentration‐time profiles for tixagevimab, cilgavimab, and AZD7442 following a single IV dose of 600 mg AZD7442 are shown in Figure 1. The PK profiles of tixagevimab and cilgavimab were largely overlapping. Maximum serum concentrations of the mAbs were rapidly achieved by the end of the IV infusion, and thereafter serum concentrations decreased in an apparent biphasic manner: a rapid decline in the first few days followed by a slow elimination phase. The estimated half‐lives of tixagevimab and cilgavimab were approximately 85 and 80 days, respectively. Serum PK parameters of tixagevimab and cilgavimab were generally similar in the Chinese population, with less than 10% difference in the means of maximum serum concentration and area under the concentration‐time curve from time 0 to infinity (Table 1).

**Table 1:** Serum Pharmacokinetic Parameters of Tixagevimab and Cilgavimab Following IV Administration of AZD7442

	Parameters (unit)	Study D8850C00001 (Global Phase 1)	Study D8850C00005 (Japan Phase 1)	Study D8850C00007 (China Phase 1)	Study D8850C00008 (China Phase 2)^a^
Analyte	AZD7442 IV dose	300 mg (n = 9)	300 mg (n = 6)^b^	600 mg (n = 13)	600 mg (n = 202)
Tixagevimab	C_max_ (µg/mL)	54.0 (5.5)	53.9 (6.5)	118.3 (9.5)	113.4 (19.4)
	AUC_inf_ (µg•day/mL)	3709 (526.7)	4493 (781.4)	7478 (1007)	8092 (1804)
	t_1/2_λz (days)	87.1 (4.5)	95.9 (11.0)	82.4 (9.7)	85.0 (11.1)
	CL (L/day)	0.04113 (0.005411)	0.03418 (0.005471)	0.04088 (0.006200)	0.03918 (0.01023)
	V_z_ (L)	5.2 (0.6)	4.7 (0.5)	4.8 (0.4)	4.7 (1.0)
Cilgavimab	C_max_ (µg/mL)	52.0 (6.3)	53.1 (6.6)	111.6 (11.0)	106.3 (19.1)
	AUC_inf_ (µg•day/mL)	3306 (491.5)	4390 (603.0)	7072 (961.4)	7586 (1680)
	t_1/2_λz (days)	91.4 (8.2)	92.0 (7.9)	79.1 (8.9)	80.4 (9.4)
	CL (L/day)	0.04618 (0.006162)	0.03469 (0.004614)	0.04322 (0.006423)	0.04185 (0.01147)
	V_z_ (L)	6.0 (0.6)	4.6 (0.5)	4.9 (0.5)	4.8 (1.1)

Data are presented as arithmetic mean (standard deviation). ClinicalTrials.gov identifiers: Global Phase 1, NCT04507256^5^; Japan Phase 1, NCT04896541^10^; China Phase 1, NCT05437289; China Phase 2, NCT05184062. AUC_inf_, area under the serum concentration‐time curve from time 0 to infinity; C_max_, maximum serum concentration; CL, clearance; IV, intravenous; t_1/2_λz, half‐life associated with terminal slope of a semilogarithmic concentration–time curve; V_z_, volume of distribution based on terminal phase after IV administration.

^a^ Present study.

^b^ Males only.

### Neutralizing Antibodies

Neutralizing antibody titers against the SARS‐CoV‐2 ancestral variant, as measured in the live‐authentic neutralization assay, are summarized by treatment group and shown in Figure S2. All participants receiving AZD7442 exhibited more than 4‐fold increases in neutralizing antibody titer at Day 8 compared with baseline and maintained these increases to Day 361. Neutralizing antibody titers were consistently higher in the AZD7442 group compared with the placebo group from Day 8 until Day 361. There were no notable differences in neutralizing antibody titers over time by vaccination status.

### Antidrug Antibodies

ADA responses to tixagevimab, cilgavimab, and AZD7442 (combined responses to tixagevimab and cilgavimab) are summarized in Table S2. Among the participants who received AZD7442, 42 (20.8%) were TE‐ADA positive.

### SARS‐CoV‐2 qRT‐PCR and Serology Tests

There was 1 positive postbaseline SARS‐CoV‐2 qRT‐PCR test in the placebo group that met the criteria for inclusion (as samples needed to be tested at a study site to be analyzed). A numerically lower proportion of participants in the AZD7442 group than the placebo group (n = 72, 35.6%; and n = 37; 52.9%, respectively) reported a posttreatment response (negative serology result at baseline and positive at 1 or more postbaseline visits) for SARS‐CoV‐2 antigen‐specific antibodies (Table S5).

### Comparison of Observed PK in Phase 1 and Phase 2 Asian Studies Versus the Phase 1 Global Study

PK parameters were generally similar for both tixagevimab and cilgavimab between Asian and global participants (Table 1). CL and volume of distribution at steady state were numerically lower in Asian participants, and the dose‐normalized exposures (area under the concentration‐time curve from time 0 to infinity) of both mAbs were higher in Asian than in global participants. Terminal elimination half‐lives of tixagevimab and cilgavimab were extended in both Asian and global participants. Compared with endogenous immunoglobulin G molecules that have half‐lives of around 3 weeks,^13^ the mean half‐lives of tixagevimab and cilgavimab were in the range of 82‐96 days and 79‐92 days, respectively, across the study populations.

### Population PK Analysis

**Figure 2:**
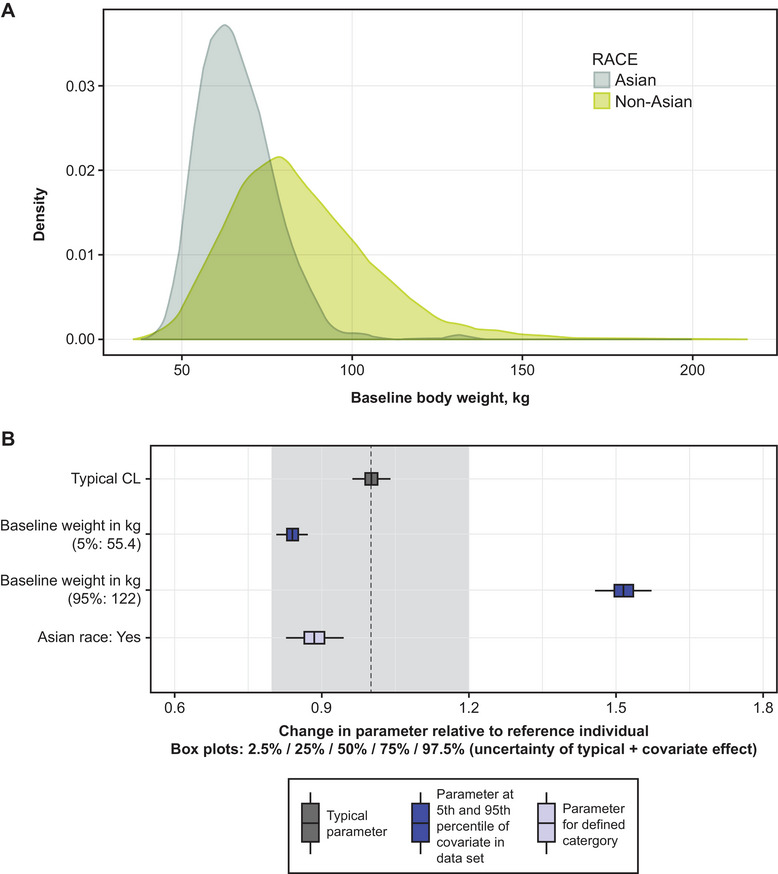
Body weight distribution for pooled population PK data set stratified by Asian race (A) and covariate effects on AZD7442 clearance (B). The AZD7442 population PK data set includes data from 8 clinical trials in global and Asian participants.^11^ In the AZD7442 population PK data set, 431 (8.72%; total n = 4940) were Asian including participants from: Japan Phase 1 (n = 30), China Phase 1 (n = 49), China Phase 2 (n = 202), and Asian participants in 5 other global studies (n = 150). In panel A, density refers to the baseline distribution of body weight stratified by race for the pooled data set including all studies. Panel B presents results of a covariate search that included Asian race as a covariate and accounted for body weight. Boxplots show the uncertainty in parameter estimation, derived from standard errors for CL and the impact of Asian race as a covariate. Boxplots represent 10,000 simulations of the typical participant and participants with body weight 55.4 kg, body weight 122 kg, and Asian race, to account for standard error in parameter estimation for CL and covariate effects. The shaded area represents the prespecified threshold of 20% for potential clinical relevance. The typical CL is for a reference individual (comparator) with following properties: baseline weight, 70 kg; female sex; age, ≤65 years; body mass index, <30 kg/m^2^; no diabetes; non‐Asian race. CL, clearance; PK, pharmacokinetic.

Effect of Asian race on CL was evaluated as a covariate using the AZD7442 population PK model.^11^ In the AZD7442 population PK data set, 431 (8.7%; n = 4940) of participants were Asian, including participants from the Japan Phase 1 study (n = 30), the China Phase 1 (n = 49) and China Phase 2 (n = 202) studies, and 5 other global studies (n = 150). In the AZD7442 population PK data set, the median body weight of Asian participants (n = 431) was approximately 65 kg, while that of non‐Asian participants (n = 4509) was approximately 82 kg (Figure 2A). A covariate search was conducted that included Asian race as a covariate in a model that accounted for body weight. Figure 2B illustrates the effect of covariates on the typical CL value. This analysis showed that Asian race was associated with an 11.6% decrease in CL compared with non‐Asian race, below the prespecified threshold of 20% for potential clinical relevance (Figure 2B).

## Discussion

The findings from this study demonstrate that a single dose of IV 600 mg AZD7442 was well tolerated over a median follow‐up of 447 days in healthy Chinese adults or those with a stable medical condition, with no safety concerns identified. Most AEs were mild or moderate in intensity, and no AESIs, infusion‐related reactions, or deaths were reported.

The most common AE in this study was COVID‐19‐related AE, with 95 of the 105 participants with COVID‐19‐related AEs having their first event during December 2022 or January 2023 when 308 or more days had passed since participants were dosed. This timing coincides with the point when COVID‐19 travel restrictions were lifted in China (December 2022), which was associated with a significant increase in reported COVID‐19 cases.^14^ AZD7442 showed an approximately 65‐fold reduction in neutralization activity against Omicron BA.4/5 subvariants.^15^ Therefore, the number of COVID‐19 cases detected in the AZD7442 group are likely related to reduced serum concentration levels (at nearly a year following dosing) combined with high exposure to resistant variants.

Overall, following IV infusion of AZD7442, tixagevimab and cilgavimab had similar PK profiles, and their estimated half‐lives of approximately 85 and 80 days, respectively, were comparable with those reported in prior studies.^4^, ^5^, ^10^ Asian race was associated with 11.6% lower CL compared with non‐Asian race in the AZD7442 population PK analysis, which included data from the present study and 7 other studies in the AZD7442 clinical program. However, as this was below the prespecified threshold of 20% for potential clinical relevance, it was not anticipated to have a clinically significant impact on serum exposure (observed to be approximately 13.1% higher) or to necessitate dose adjustment in Asian populations. The apparent difference in AZD7442 exposure between Asian and non‐Asian populations could also be attributed to lower body weight among Asian participants. Based on simulations using an AZD7442 population PK model, body weight was found to have a greater than 20% predicted effect on exposure but was not considered to have a clinically relevant impact requiring dose adjustment.^11^ These results from the AZD7442 population PK model are consistent with previous reports showing that body weight is a commonly identified significant covariate in the population PK modeling of mAbs, and that race is seldom found to be a significant covariate for mAb PK after accounting for body weight.^16^, ^17^ Lower body weight is usually associated with a decreased CL and smaller volume of distribution thus a higher exposure compared to larger body weight.

The sudden increase in neutralizing antibody titer levels at Day 361 in placebo‐treated participants is likely due to the relaxation of COVID‐19 restrictions in China during December 2022, as mentioned above, resulting in an increase of COVID‐19 infections late in the study due to Omicron BA.5 subvariants,^14^, ^18^ which could evade both vaccine‐induced and natural immunity to prior SARS‐CoV‐2 infections, as well as mAbs.^15^ Participants who were vaccinated or had COVID‐19 infections during the study were not excluded from the PD analysis set; hence, this may have had an impact on the neutralizing antibody titers from infections or vaccinations in both treatment groups.

Overall, median ADA titers against AZD7442 (combined responses) in TE‐ADA‐positive participants were numerically similar between AZD7442 and placebo groups. The presence of ADAs had no apparent effect on PK or safety. The overall immunogenicity profile of AZD7442 was generally similar between Chinese participants from this study and Japanese and global participants from prior studies: ADA prevalence and incidence were numerically similar, the majority of ADA‐positive participants were not classified as TE‐ADA positive, and the medians of maximum ADA titers for AZD7442 in TE‐ADA–positive participants were generally low and similar.^5^, ^10^, ^19^, ^20^

Although this study was not powered to assess clinical efficacy, a numerically lower proportion of participants in the AZD7442 group reported a posttreatment response for SARS‐CoV‐2 antigen‐specific antibodies over the first 361 days of the study compared with the placebo group in the SARS‐CoV‐2 serology test, suggesting that IV 600 mg AZD7442 had a protective effect. Findings from real‐world studies in immunocompromised patients also supported a protective effect of AZD7442 against Omicron variants. A meta‐analysis of real‐world studies conducted during June 2021 to October 2022 (dominated by Omicron variants including BA.1, BA.2, and BA.4/5) suggested that preexposure prophylaxis with AZD7442 provided reductions in breakthrough infections of 40.5% and reductions in hospitalizations, intensive care unit admissions, and COVID‐19‐related mortality of 66.2%, 82.1%, and 92.4%, respectively.^21^ However, later in vitro studies showed loss of AZD7442 neutralization against subsequently emerging SARS‐CoV‐2 variants, including BQ1.1 and XBB.^8^, ^9^

Limitations of this study include a low proportion of participants that were aged greater than 60 years, mainly due to prioritization of healthy participants and those with stable medical conditions. Furthermore, antibody titers were performed only against the SARS‐CoV‐2 ancestral variant, which is no longer in circulation.

This Phase 2 study showed that IV 600 mg AZD7442 was well tolerated in Chinese adult participants, with PK characteristics consistent with previous studies in global and Japanese populations.^5^, ^10^ Population PK analysis of AZD7442 indicated that Asian race was not a clinically significant covariate on mAb CL, and that no dose adjustment would be required. The findings reported in this study provide useful information to inform the development of future long‐acting mAbs utilizing YTE and TM modifications.

## Author Contributions

All authors meet the ICMJE authorship criteria.

## Conflicts of Interest

W.Z. and J.Z. received investigator fees from AstraZeneca for the conduct of the study. Y.Z., S.L., Ha.Z., O.S., W.T., and Hu.Z. are employees of, and may hold stock and/or stock options in, AstraZeneca. X.G., Y.L., and C.C.C. were employees of AstraZeneca at the time of the study.

## Funding

This work was supported by AstraZeneca.

## Supporting information



Supporting information

## Data Availability

Data underlying the findings described in this manuscript may be requested in accordance with AstraZeneca's data sharing policy described at https://astrazenecagrouptrials.pharmacm.com/ST/Submission/Disclosure. AstraZeneca Group of Companies allows researchers to submit a request to access anonymized participant‐level clinical data, aggregate clinical or genomics data (when available), and anonymized clinical study reports through the Vivli web‐based data request platform.
