# Supplementary material for: Safety and Pharmacokinetics of Long‐Acting Monoclonal Antibodies Tixagevimab and Cilgavimab (AZD7442) in a China Phase 2 Study and Evaluation of Asian Race Effect
Source: Clin Pharmacol Drug Dev. 2025 Sep 5;14(11):846–55. doi: 10.1002/cpdd.1586 (PMC12583982; doi:10.1002/cpdd.1586)
Supplement: Supplementary file 1 — Supporting information [file CPDD-14-846-s001.pdf]

Supplementary Materials

Inclusion criteria

For inclusion in the study, participants had to fulfill all the following criteria:

Age

1 Chinese adults ≥18 years of age, at the time of signing the informed consent.

Informed consent

2 Capable of giving signed informed consent, which included compliance with the requirements and restrictions listed in the informed consent form and in the protocol.

Type of participant and disease characteristics

3 Healthy participants or participants with stable medical conditions.

(a) Participants who were healthy by medical history, physical examination, and baseline safety laboratory tests, as judged by the investigator.

OR

(b) Participants with stable disease/condition, as judged by the investigator, who could benefit from passive immunization with antibodies. “Stable” was defined below as:

No hospitalization or emergency visit for worsening of disease/condition within the 12 months prior to enrollment.

No acute change in the participant's condition at the time of study enrollment, and, as judged by the investigator, no exacerbation in disease/condition and no significant change in therapy expected during at least the first 6 months of the study.

Participation in the clinical study not expected to pose a significant risk to the participant, as judged by the investigator.

Diseases/conditions include those who are elderly (≥60 years of age) or obese (body mass index ≥30 kg/m^2), or those who have chronic obstructive pulmonary disease, congestive heart failure (New York Heart Association classification ≤class II), chronic kidney disease, chronic liver disease, immunocompromised state requiring maintenance use of corticosteroids and/or other immunosuppressive medicines, intolerant of vaccines, or another disease as judged by the investigator.

4 Negative results of severe acute respiratory syndrome coronavirus 2 (SARS-CoV-2) quantitative reverse transcription-polymerase chain reaction (qRT-PCR) test within 14 days prior to randomization. NOTE: A negative SARS-CoV-2 qRT-PCR retest result was required if a participant had symptoms of infection or had any known/suspected exposure after the initial test.

5 Able to complete the follow-up period through Day 451 as required by the Clinical Study Protocol.

Reproduction

Contraceptive use by males or females was consistent with local regulations regarding the methods of contraception for those participating in clinical studies.

6 Contraceptive use by males or females:

(a) Male participants: To avoid transfer of fluids to a sexual partner, all male participants were required to use a condom from Day 1 and to agree to continue through 365 days following administration of the study intervention.

(b) Female participants: Female participants of childbearing potential were required to use 1 highly effective form of birth control. Females of childbearing potential who were sexually active with a non-sterilized male partner were required to agree to use 1 highly effective method of birth control, as defined below, from Day 1 and to agree to continue through 365 days following administration of the study intervention. Cessation of contraception after this point was to be discussed with a responsible physician. Periodic abstinence (calendar, symptothermal, post-ovulation methods), withdrawal (coitus interruptus), spermicides only, and lactational amenorrhea method were not acceptable methods of contraception. Female condom and male condom were not to be used together. All females of childbearing potential were to have a negative serum pregnancy test result at Visit 1 and throughout the study as indicated per the schedule of activities.

Females not of childbearing potential were defined as females who were either permanently sterilized (hysterectomy, bilateral oophorectomy, or bilateral salpingectomy), or who were postmenopausal. Females were considered postmenopausal if they had been amenorrheic for 12 months prior to the planned date of randomization without an alternative medical cause. The following age-specific requirements applied:

Females <50 years of age were considered postmenopausal if they had been amenorrheic for 12 months or more following cessation of exogenous hormonal treatment and follicle stimulating hormone levels in the postmenopausal range.

Females ≥50 years of age were considered postmenopausal if they had been amenorrheic for 12 months or more following cessation of all exogenous hormonal treatment.

A highly effective method of contraception was defined as one that could achieve a failure rate of <1% per year when used consistently and correctly

Exclusion criteria

Any of the following were regarded as a criterion for exclusion from the study:

Medical conditions

1 Known history of allergy or reaction to any component of the study intervention formulation.

2 Previous hypersensitivity, infusion-related reactions, or severe adverse reaction following administration of a monoclonal antibody (mAb).

3 Significant infection or other acute illness, including fever >100°F (>37.8°C) on the day prior to or day of randomization. Participants excluded for transient acute illness could be dosed if illness resolved within the 28-day screening period. Otherwise, the participant was to be reported as a screen failure. Upon obtaining an informed consent again, the participant could be rescreened just once.

4 History of infection with severe acute respiratory syndrome or Middle East respiratory syndrome.

5 History of laboratory-confirmed SARS-CoV-2 infection. NOTE: Participants with any positive SARS-CoV-2 qRT-PCR result based on available data at screening were excluded. Unvaccinated participants with any positive SARS-CoV-2 serology result based on available data at screening were excluded.

6 Any clinical signs and symptoms consistent with coronavirus disease 2019 (COVID-19), eg, fever, dry cough, dyspnea, sore throat, fatigue, or confirmed infection by appropriate laboratory test within the last 4 weeks prior to screening or randomization.

7 History of malignancy.

(a) Participants who had a history of basal cell carcinoma, localized squamous cell carcinoma of the skin, or in situ carcinoma of the cervix were eligible provided that the participant was in remission and curative therapy was completed at least 12 months prior to enrollment.

(b) Participants who had a history of other malignancies were eligible provided that the participant was in remission and curative therapy was completed ≥5 years prior to enrollment.

8 History of clinically significant bleeding disorder (eg, factor deficiency, coagulopathy, or platelet disorder), or prior history of significant bleeding or bruising following intramuscular injections or venepuncture.

9 Current active liver disease.

Chronic stable hepatitis B and C (including positive testing for hepatitis B surface antigen or hepatitis C antibody), or other stable chronic liver disease were acceptable if the participant otherwise met eligibility criteria. Stable chronic liver disease was generally defined by the absence of ascites, encephalopathy,

coagulopathy, hypoalbuminemia, esophageal or gastric varices, or persistent jaundice, or cirrhosis.

10 History of human immunodeficiency virus (HIV) or positive for HIV at screening.

11 History of alcohol or drug abuse within the past 2 years that, according to the investigator, might have affected assessments of safety or ability of the participant to comply with all study requirements.

12 Any other significant disease, disorder, or finding that might have significantly increased the risk to the participant because of participation in the study, affected the ability of the participant to participate in the study, or impaired interpretation of the study data.

Significant laboratory abnormalities

13 Any of the following laboratory abnormalities at screening:

(a) Aspartate transaminase or alanine transaminase >2.0 × upper limit of normal (ULN), alkaline phosphatase >1.5 × ULN, or total bilirubin >1.5 × ULN (unless due to Gilbert's syndrome)

(b) Serum creatinine >176 µmol/L (2 mg/100 mL)

(c) Hemoglobin <10 g/dL (100 g/L)

(d) Platelet count <100 × 10^3/µL (100 × 10^9/L)

(e) White blood cell count <3.5 × 10^3/µL (3.5 × 10^9/L) or neutrophil count <1.5 × 10^3/µL (1.5×10^9/L)

(f) Any other laboratory value in the screening panel that, in the opinion of the investigator, was clinically significant or might have confounded analysis of study results.

Prior/concomitant therapy

14 Any newly initiated drug therapy within 7 days or 5 drug half-lives (whichever was longer) prior to screening.

15 Receipt of immunoglobulin or blood products within 6 months prior to screening.

16 Any prior receipt of an investigational or licensed mAb/biologic indicated for the prevention of SARS-CoV-2 or COVID-19 or was scheduled to receive any investigational or licensed mAb/biologic indicated for the prevention of SARS-CoV-2 or COVID-19.

17 Receipt of any COVID-19 vaccine within 6 months prior to randomization or scheduled to receive any COVID-19 vaccine.

Prior/concurrent clinical study experience

18 Receipt of any investigational product within 90 days or 5 antibody half-lives (whichever was longer) prior to Day 1 or expected receipt of investigational product during the follow-up period, or concurrent participation in another interventional study.

Other exclusions

19 Involvement in the planning and/or conduct of the study (applied to both AstraZeneca staff and/or staff at the study site).

20 Judgment by the investigator that the participant should not participate in the study if the participant was unlikely to comply with study procedures, restrictions, and requirements.

21 For females only: currently pregnant (confirmed with a positive pregnancy test) or breastfeeding.

Table S1. Study Sites and Institutional Review Boards/Ethics Committees

Institution	Responsible Institutional Review Board/Ethics Committee
Huashan Hospital, Fudan University, Shanghai	Institutional Review Board Huashan Hospital, Fudan
Xiangya Hospital Central South University, Changsha	Medical Ethics Committee of Xiangya Hospital Central South University
The Affiliated Hospital of Xuzhou Medical University, Xuzhou	Medical Ethics Committee of the Affiliated Hospital of Xuzhou Medical University
Affiliated Hospital of Hebei University, Baoding	Ethics Committee of Affiliated Hospital of Hebei University
The First Affiliated Hospital of Chongqing Medical University, Chongqing	Ethical Committee for Drug Clinical Trials, The First Affiliated Hospital of Chongqing Medical University
Lanzhou University Second Hospital, Gansu	Ethical Committee for Clinical Drug Trials of Lanzhou University Second Hospital
Shuguang Hospital affiliated to Shanghai University of Traditional Chinese Medicine, Shanghai	IRB of Shuguang Hospital affiliated with Shanghai University of Traditional Chinese Medicine
The First Affiliated Hospital of Fujian Medical University, Fuzhou	Clinical Trial Branch of Medical Ethics Committee of the First Affiliated Hospital of Fujian Medical University
Hainan General Hospital, Haikou	Medical Ethics Committee of Hainan General Hospital
The Second Affiliated Hospital of Soochow University, Suzhou	The Ethics Committee of the Second Hospital of Soochow University
The First Affiliated Hospital of Xinjiang Medical University, Urumqi	Ethics Committee of the First Affiliated Hospital of Xinjiang Medical University
Peking University First Hospital, Beijing	Biomedical Research Ethics committee of Peking University First Hospital
Yueyang Hospital of Intergrated Traditional Chinese and Western Medicine, Shanghai University of Traditional Chinese Medicine, Shanghai	Ethics Committee of Yueyang Hospital of Integrated Traditional Chinese and Western Medicine, Shanghai University of Traditional Chinese Medicine
Hangzhou First People's Hospital, Hangzhou	Ethics Committee of Hangzhou First People's Hospital

Table S2. Summary of ADA Responses to Tixagevimab, Cilgavimab, and AZD7442 (Tixagevimab/Cilgavimab) During the Study (Tixagevimab, Cilgavimab, and AZD7442 ADA Evaluable Analysis Sets)

ADA category	Statistic	Tixagevimab		Cilgavimab		AZD7442	
		AZD7442 600 mg IV N = 202	Placebo N = 70	AZD7442 600 mg IV N = 202	Placebo N = 70	AZD7442 600 mg IV N = 202	Placebo N = 70
ADA positive at baseline and/or post-baseline (ADA prevalence)	n (%)	44 (21.8)	8 (11.4)	39 (19.3)	5 (7.1)	54 (26.7)	11 (15.7)
	Median (min., max.)	320.0 (80, 5120)	320.0 (80, 1280)	160.0 (40, 2560)	80.0 (40, 2560)	320.0 (80, 5120)	320.0 (40, 2560)
TE-ADA positive (ADA incidence)[a-c]	n (%)	35 (17.3)	1 (1.4)	30 (14.9)	3 (4.3)	42 (20.8)	3 (4.3)
	Median (min., max.)	320.0 (160, 5120)	160.0 (160, 160)	320.0 (80, 2560)	160.0 (80, 2560)	320.0 (80, 5120)	160.0 (80, 2560)
Treatment-induced ADA positive[a-c]	n (%)	35 (17.3)	1 (1.4)	29 (14.4)	1 (1.4)	42 (20.8)	2 (2.9)
	Median (min., max.)	320.0 (160, 5120)	160.0 (160, 160)	320.0 (80, 2560)	80.0 (80, 80)	320.0 (80, 5120)	120.0 (80, 160)
Treatment-boosted ADA positive[a-c]	n (%)	0	0	1 (0.5)	2 (2.9)	1 (0.5)	2 (2.9)
	Median (min., max.)	NA (NA, NA)	NA (NA, NA)	640.0 (640, 640)	1360.0 (160, 2560)	1280.0 (1280, 1280)	1360.0 (160, 2560)
Non–TE-ADA positive[a-c]	n (%)	9 (4.5)	7 (10.0)	9 (4.5)	2 (2.9)	12 (5.9)	8 (11.4)
	Median (min., max.)	160.0 (80, 640)	320.0 (80, 1280)	80.0 (40, 2560)	60.0 (40, 80)	120.0 (80, 2560)	320.0 (40, 640)
Both baseline and post-baseline positive	n (%)	2 (1.0)	6 (8.6)	4 (2.0)	3 (4.3)	6 (3.0)	8 (11.4)
	Median (min., max.)	400.0 (160, 640)	320.0 (320, 1280)	400.0 (40, 2560)	160.0 (80, 2560)	480.0 (160, 2560)	320.0 (80, 2560)
Only baseline positive	n (%)	7 (3.5)	0	5 (2.5)	1 (1.4)	8 (4.0)	1 (1.4)
	Median (min., max.)	160.0 (80, 640)	NA (NA, NA)	80.0 (80, 80)	40.0 (40, 40)	80.0 (80, 160)	40.0 (40, 40)
ADA persistently positive[d-f]	n (%)	30 (14.9)	0	29 (14.4)	1 (1.4)	37 (18.3)	1 (1.4)
	Median (min., max.)	480.0 (160, 5120)	NA (NA, NA)	320.0 (80, 2560)	2560.0 (2560, 2560)	640.0 (80, 5120)	2560.0 (2560, 2560)
	n (%)	5 (2.5)	1 (1.4)	1 (0.5)	2 (2.9)	5 (2.5)	2 (2.9)

ADA transiently positive[d–f]	Median (min., max.)	160.0 (160, 320)	160.0 (160, 160)	320.0 (320, 320)	120.0 (80, 160)	160.0 (160, 320)	120.0 (80, 160)

If a participant has >1 non-missing titer during the study, the maximum titer for each participant is summarized. MRD for tixagevimab = 80; MRD for cilgavimab = 40. ADA, anti-drug antibody; IV, intravenous; max., maximum; min., minimum; MRD, minimum required dilution; n, the number of participants satisfying the conditions of the specified ADA category; N, the number of participants in each respective column among the ADA Evaluable Analysis Set; NA, not applicable; TE, treatment-emergent.

[a]TE-ADA positive to tixagevimab is defined as the sum of treatment-induced ADA positive (ADA negative at baseline and post-baseline ADA positive with ADA titer ≥160) and treatment-boosted ADA positive (ADA positive at baseline and boosted the pre-existing titer during the study period by ≥4-fold). Non–TE-ADA positive to tixagevimab is defined as ADA positive to tixagevimab but not fulfilling the definition of TE-ADA positive. ADA incidence is the proportion of TE-ADA–positive participants in a population.

[b]TE-ADA positive to cilgavimab is defined as the sum of treatment-induced ADA positive (ADA negative at baseline and post-baseline ADA positive with ADA titer ≥80) and treatment-boosted ADA positive (ADA positive at baseline and boosted the pre-existing titer during the study period by ≥4-fold). Non–TE-ADA positive to cilgavimab is defined as ADA positive to cilgavimab but not fulfilling the definition of TE-ADA positive. ADA incidence is the proportion of TE-ADA–positive participants in a population.

[c]TE-ADA positive to AZD7442 is defined as TE-ADA positive to tixagevimab and/or cilgavimab. Non–TE-ADA positive to AZD7442 is defined as ADA positive to tixagevimab and/or cilgavimab, but not fulfilling the definition of TE-ADA positive. ADA incidence is the proportion of TE-ADA–positive participants in a population.

[d]ADA persistently positive to tixagevimab is defined as being TE-ADA positive and having ≥2 ADA post-baseline positive assessments with titer ≥160 (with ≥16 weeks between the first and last positive assessment) or positive with ADA titer ≥160 at the last post-baseline assessment. ADA transiently positive is defined as being TE-ADA positive and having ≥1 post-baseline ADA-positive assessment with titer ≥160, and not fulfilling the conditions of persistently positive.

[e]ADA persistently positive to cilgavimab is defined as being TE-ADA positive and having ≥2 ADA post-baseline positive assessments with titer ≥80 (with ≥16 weeks between first and last positive assessment) or positive with ADA titer ≥80 at last post-baseline assessment. ADA transiently positive is defined as being TE-ADA positive and having ≥1 post-baseline ADA-positive assessment with titer ≥80, and not fulfilling the conditions of persistently positive.

[f]ADA persistently positive to AZD7442 is defined as persistently positive to tixagevimab and/or cilgavimab. ADA transiently positive is defined as being TE-ADA positive to AZD7442, and not fulfilling the conditions of persistently positive.

Table S3. Baseline and Demographic Characteristics (Safety Analysis Set [Final Analysis])

	AZD7442 600 mg IV<br>N = 202	Placebo<br>N = 70
Age, years, mean (SD)	33.7 (11.7)	35.7 (12.1)
18-44 years, n (%)	170 (84.2)	54 (77.1)
45-60 years, n (%)	20 (9.9)	12 (17.1)
>60 years, n (%)	12 (5.9)	4 (5.7)
Sex, n (%)		
Male	140 (69.3)	48 (68.6)
Female	62 (30.7)	22 (31.4)
Race, n (%)		
Asian	202 (100)	70 (100)
Ethnicity, n (%)		
Not Hispanic or Latino	202 (100)	70 (100)
BMI, kg/m^2, mean (SD)	23.8 (3.1)	23.9 (3.0)
COVID-19 vaccination ≤6 months prior to randomization, n (%)	104 (51.5)	36 (51.4)

BMI (kg/m^2) = weight (kg)/height (m)^2. BMI, body mass index; COVID-19, coronavirus disease 2019; IV, intravenous; n, number of participants in analysis for a continuous variable and number of participants per category for a categorical variable; N, number of participants per treatment group; SD, standard deviation.

Table S4. Overall Summary of AEs (Safety Analysis Set [Final Analysis])

	AZD7442 600 mg IV<br>N = 202	Placebo<br>N = 70
	n (%)[a]	n (%)[a]
Any AE	147 (72.8)	56 (80.0)
COVID-19 relevant AE	71 (35.1)	34 (48.6)
Most common AEs (>5% in either group)		
COVID-19	58 (28.7)	20 (28.6)
Upper respiratory tract infection	29 (14.4)	13 (18.6)
Influenza-like illness	20 (9.9)	4 (5.7)
Protein urine present	15 (7.4)	6 (8.6)
Suspected COVID-19	12 (5.9)	14 (20.0)
Headache	5 (2.5)	4 (5.7)
Nasopharyngitis	3 (1.5)	4 (5.7)
Any SAE	6 (3.0)	3 (4.3)
Deaths	0	0
Any AE leading to dose interruption	2 (1.0)	0
Any AE leading to withdrawal from study	0	0
Any AESI	0	0
Any drug-related AE[b]	19 (9.4)	7 (10.0)
Any drug-related SAE[b]	0	0

The table includes AEs with an onset date/time on or after the date/time of first dose of investigational medicinal product. AE, adverse event; AESI, AE of special interest; COVID-19, coronavirus disease 2019; IV, intravenous; N, number of participants per treatment group; n, number of participants per category; SAE, serious AE.

[a]Participants with multiple events in the same category were counted only once in that category. Participants with events in >1 category were counted once in each of those categories.

[b]Drug-related, as assessed by investigator.

Table S5. SARS-CoV-2 Serology Test Results (Full Analysis Set)

		AZD7442 600 mg IV N = 202	Placebo N = 70	Total N = 272
Post-treatment response, n (%)[a]		72 (35.6)	37 (52.9)	109 (40.1)
Baseline	Positive	25 (12.4)	5 (7.1)	30 (11.0)
	Negative	156 (77.2)	59 (84.3)	215 (79.0)
	Missing	21 (10.4)	6 (8.6)	27 (9.9)
Day 181	Positive	18 (8.9)	4 (5.7)	22 (8.1)
	Negative	158 (78.2)	60 (85.7)	218 (80.1)
	Missing	26 (12.9)	6 (8.6)	32 (11.8)
Day 361	Positive	92 (45.5)	39 (55.7)	131 (48.2)
	Negative	83 (41.1)	24 (34.3)	107 (39.3)
	Missing	27 (13.4)	7 (10.0)	34 (12.5)

IV, intravenous; n, number of participants per category; N, number of participants per treatment group.

[a] A post-treatment response is defined as a negative serology result at baseline and positive at ≥1 post-baseline. All available serology data prior to coronavirus disease 2019 vaccination (if applicable) during the study were included in evaluation.

Figure S1. Participant disposition.

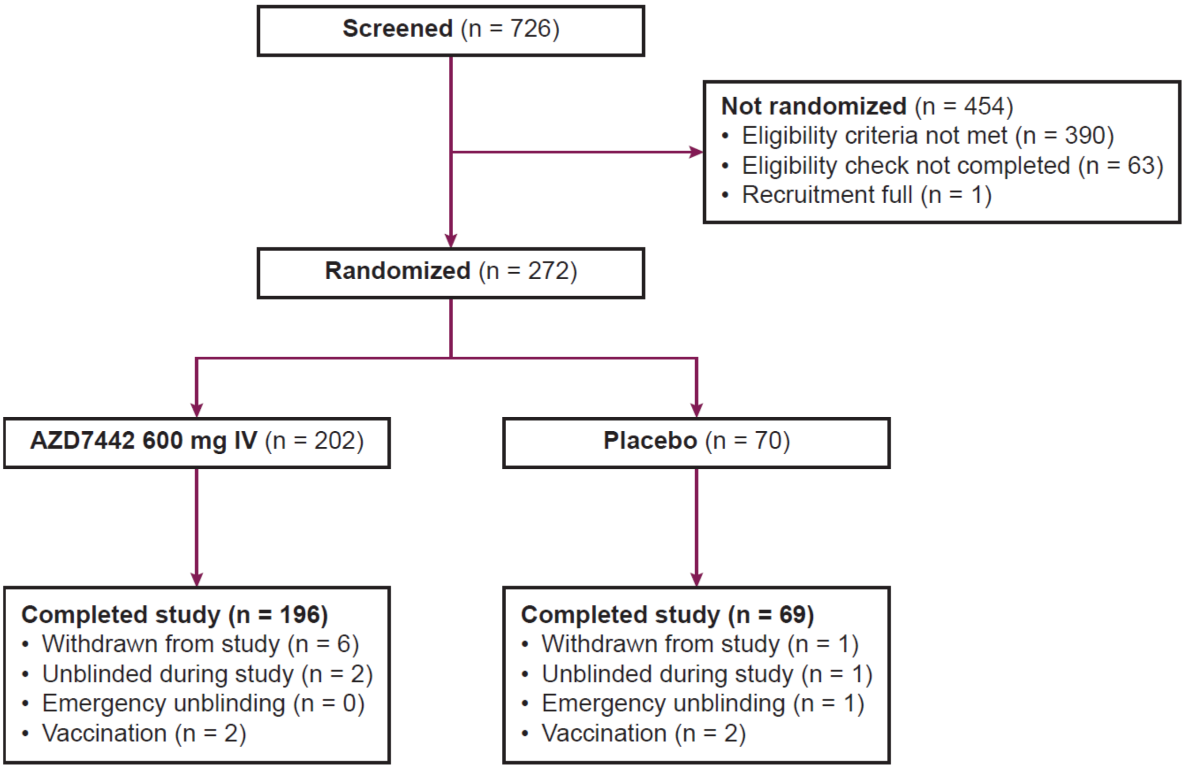

IV, intravenous; n, number of participants per category.

Figure S2. Box plot of neutralizing antibody titers against severe acute respiratory syndrome coronavirus 2 over time (Pharmacodynamic Analysis Set).

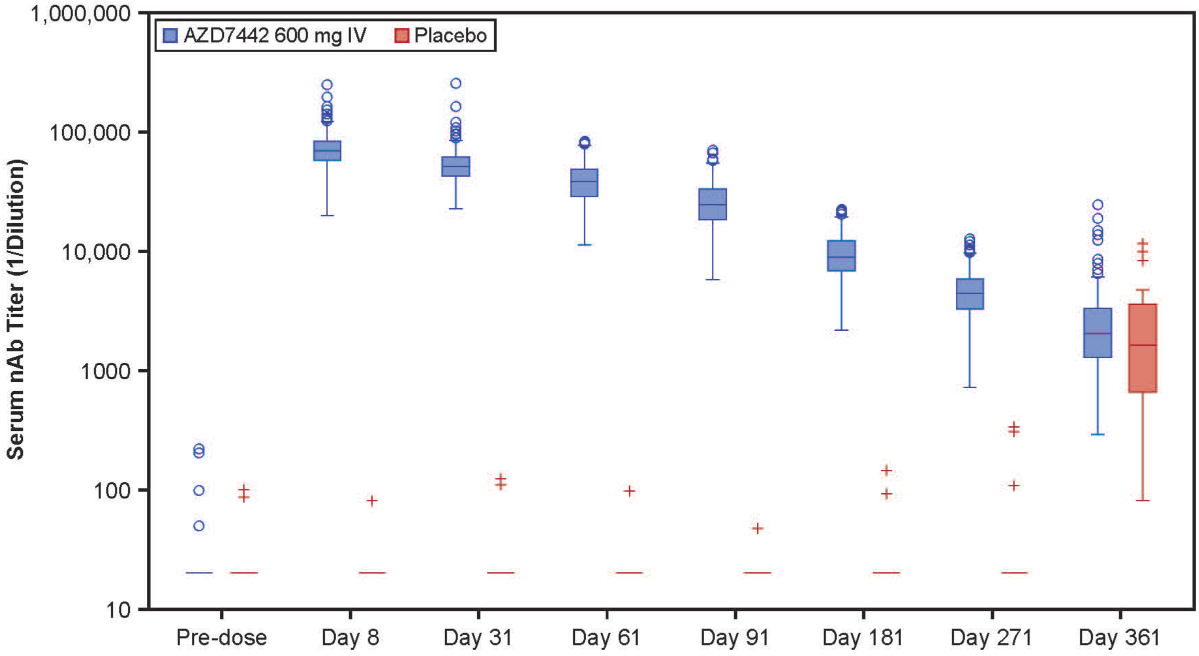

Serum nAb titers were reported as the reciprocal of the serum dilution conferring 50% inhibition of pseudovirus infection. MRD = 40. For titers that reported less than the MRD, a value of 20 was imputed in the calculation; for such results, only a median line (instead of a box) is shown. The bottom and top edges of the boxes are the 25th (Q1) and 75th (Q3) percentiles (the difference is the interquartile range), and the lines inside the boxes is the median value. The whiskers that extend from the boxes indicate the minimum and maximum values within the range of 1.5×interquartile range (Q3-Q1). The circles or + signs represent outliers outside of this range. IV, intravenous; MRD, minimum required dilution.
